# Expression and purification of a native Thy1-single-chain variable fragment for use in molecular imaging

**DOI:** 10.1038/s41598-021-02445-2

**Published:** 2021-11-29

**Authors:** Natacha Jugniot, Rakesh Bam, Ramasamy Paulmurugan

**Affiliations:** Department of Radiology, Molecular Imaging Program at Stanford (MIPS), Canary Center for Cancer Early Detection at Stanford, Stanford University School of Medicine, Stanford University, 3155 Porter Drive, Palo Alto, CA 94304 USA

**Keywords:** Cancer, Translational research, Preclinical research

## Abstract

Molecular imaging using singlechain variable fragments (scFv) of antibodies targeting cancer specific antigens have been considered a non-immunogenic approach for early diagnosis in the clinic. Usually, production of proteins is performed within *Escherichia coli.* Recombinant proteins are either expressed in *E. coli* cytoplasm as insoluble inclusion bodies, that often need cumbersome denaturation and refolding processes, or secreted toward the periplasm as soluble proteins that highly reduce the overall yield. However, production of active scFvs in their native form, without any heterologous fusion, is required for clinical applications. In this study, we expressed an anti-thymocyte differentiation antigen-scFv (Thy1-scFv) as a fusion protein with a N-terminal sequence including 3 × hexa-histidines, as purification tags, together with a Trx-tag and a S-tag for enhanced-solubility. Our strategy allowed to recover ~ 35% of Thy1-scFv in the soluble cytoplasmic fraction. An enterokinase cleavage site in between Thy1-scFv and the upstream tags was used to regenerate the protein with 97.7 ± 2.3% purity without any tags. Thy1-scFv showed functionality towards its target on flow cytometry assays. Finally, in vivo molecular imaging using Thy1-scFv conjugated to an ultrasound contrast agent (MB_Thy1-scFv_) demonstrated signal enhancement on a transgenic pancreatic ductal adenocarcinoma (PDAC) mouse model (3.1 ± 1.2 a.u.) compared to non-targeted control (0.4 ± 0.4 a.u.) suggesting potential for PDAC early diagnosis. Overall, our strategy facilitates the expression and purification of Thy1-scFv while introducing its ability for diagnostic molecular imaging of pancreatic cancer. The presented methodology could be expanded to other important eukaryotic proteins for various applications, including but not limited to molecular imaging.

## Introduction

Molecular imaging techniques play a central role in clinical oncology by enhancing diagnostic and therapeutic approaches. Ultrasound (US) molecular imaging (USMI) is a recent modality with greatly improved detection accuracy compared to conventional US. When using molecularly targeted US contrast agents, USMI has shown combined advantages of US modality and molecular imaging. With the recent first molecular US contrast agent to enter clinical trials, innovation in human cancer imaging by USMI lies ahead. Targeted US contrast agents called ‘microbubbles’ (MBs) are gas-filled microparticles synthesized by conjugating specific ligands onto the MB shell, making them to bind tumor neovascular targets thus enabling enhanced-tumor detection. However, producing targeted-ligands as recombinant proteins/antibodies without heterologous tags is needed to generate non-immunogenic agents for translational applications.

With the introduction of recombinant DNA technology in 1974^1^, faster, easier, and more efficient production of heterologous proteins have been performed compared to purifying proteins from their natural sources. Nowadays, diverse expression systems such as bacteria, yeast, insect cells, mammalian cells, cell-free systems, transgenic animals and plants have been used for the expression of recombinant proteins. Choice in host system plays a critical role not only in the expression of the protein of interest but also in the way it can be subsequently purified^2^.

**Figure 1:**
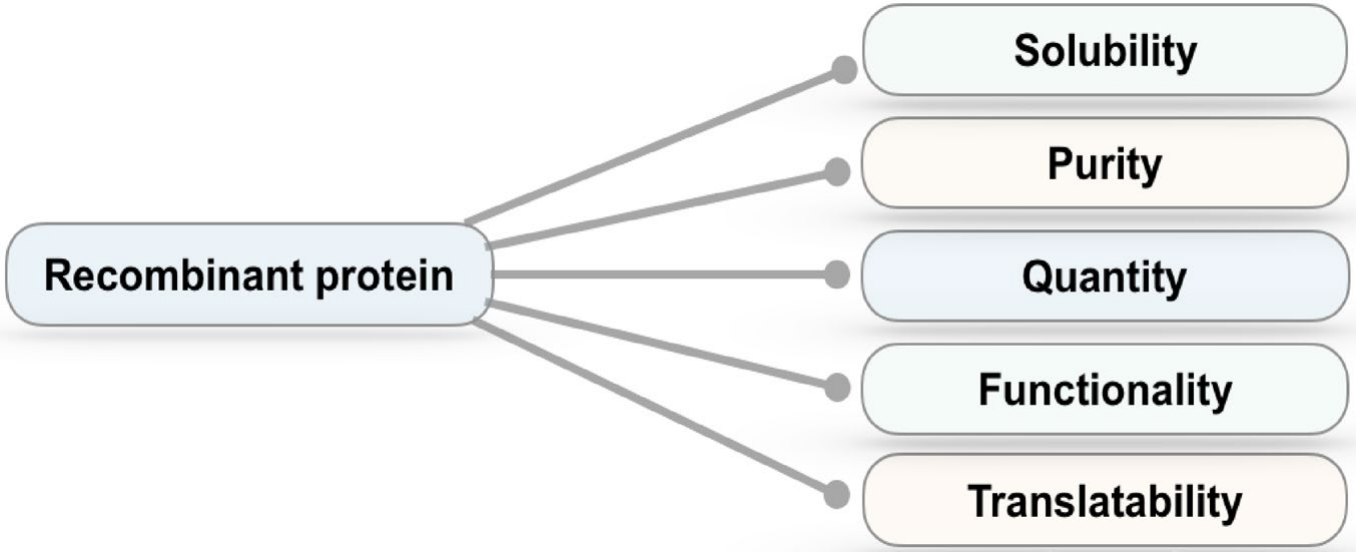
Recombinant protein checkpoints.

*Escherichia coli * remains the most attractive host for recombinant protein expression^3,4^. However, as a prokaryotic system, *E. coli* may not be able to produce some eukaryotic proteins in their native form^5^. To overcome such issues, strategies include the use of mutated host strains, mRNA enhanced-stability, use of optimal codons, co-expression of molecular chaperones such as foldases and post-translational protein modifying enzymes, as well as optimization of growth conditions^6,7^. Despite plethora of methodologies available, challenges remain in providing recombinant proteins with relevant quantity, purity, solubility, functionality, and translatability toward clinical applications (Fig. 1). Periplasmic protein expression is considered a favorable approach for disulfide bond formation in *E. coli,* and was initially the most frequently used methodology^8^. Alternatively, expressing proteins in the cytoplasm leads to much higher expression levels but results in aggregation into insoluble inclusion bodies (IBs)^9,10^. Although useful in many cases where large amounts of proteins are needed, denaturation/refolding protocols of IBs necessitate the use of strong denaturants and reducing agents^11^ which can lead to improper refolding and might not give full recovery of the protein with its biological activity^12^. On the other hand, a wide range of fusion partners with solubilization tags (e.g*.,* thioredoxin (Trx), poly(NANP) (N-acetylneuraminic acid phosphatase), S-tag) are now available for enhanced expression of cytosoluble recombinant proteins, and affinity tags (e.g., maltose binding protein (MBP), glutathione-S-transferase (GST), hexa-histidine tag) for more efficient purification processes^13,14^. Although tag sequences are essential for the expression and purification of recombinant proteins, they can interfere with the structure and function of their fusion partner while limiting their application in the clinic due to immunogenicity. Therefore, tag removal should be considered, especially if the protein of interest is intended for clinical applications or structural studies^15^. Hence, developing protocols for the recombinant production of eukaryotic proteins in their native form is important.

**Figure 2:**
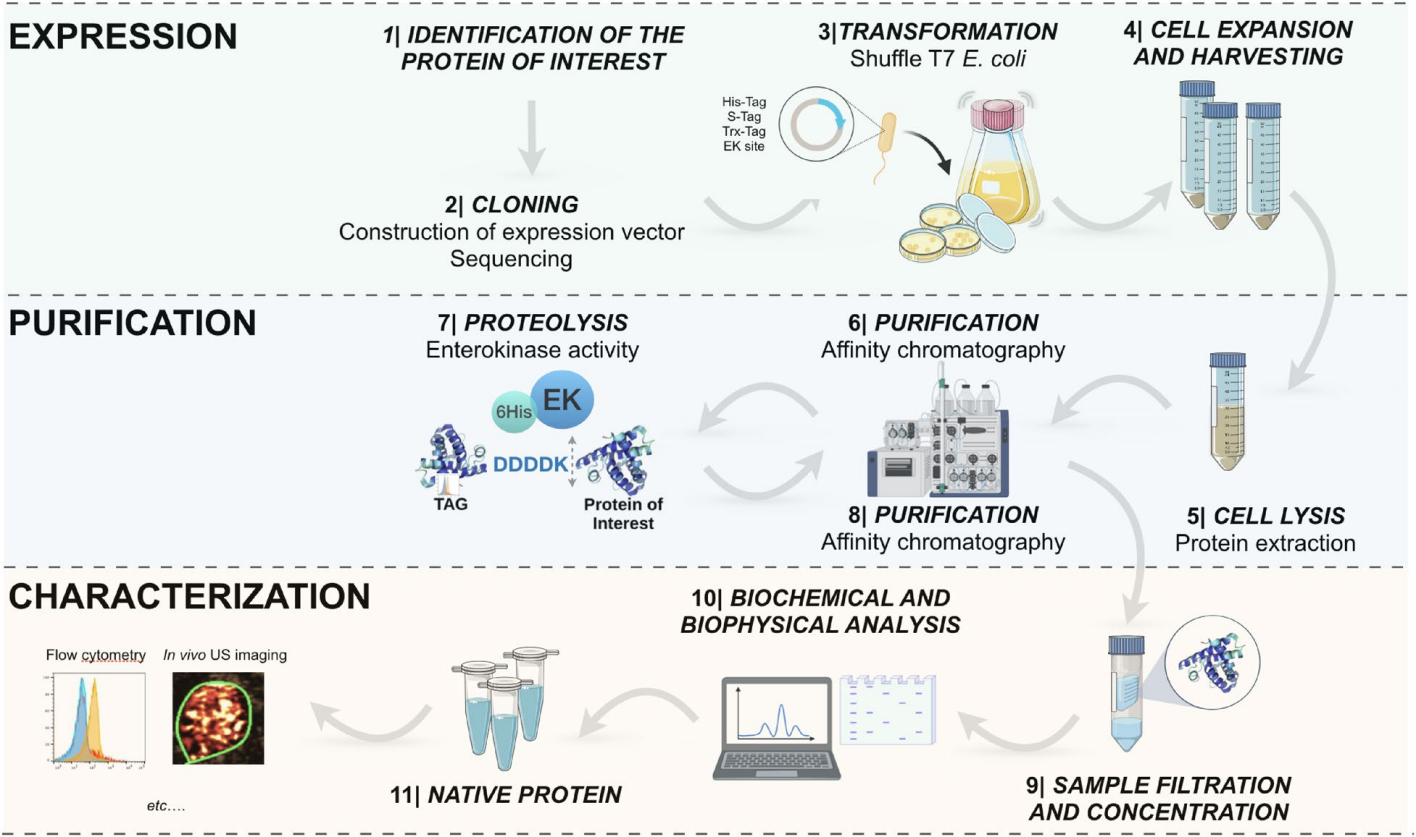
Schematic workflow showing the methodology employed for the construction, expression and purification of recombinant protein in its native form.

With the goal of developing US contrast agents for pancreatic ductal adenocarcinoma (PDAC) imaging, we chose to express a single-chain variable fragment (scFv) targeting the thymocyte differentiation antigen (Thy1/CD90)^16^. Thy1 is a cell-surface glycoprotein originally described as a mouse thymocyte differentiation marker. Subsequently, Thy1 has been shown to be expressed in other tissues, including overexpression in the neovasculature of various human cancers (e.g., colon cancer^17^, glioblastoma^18^, hepatocellular carcinoma^19^, ovarian cancer^20^, prostate cancer^21^ and PDAC^22,23^). Proteomic and immunohistochemical analysis in human tissue samples revealed Thy1 as a molecular marker upregulated on the neovasculature of PDAC^22,23^. Importantly, 81% of PDAC cases stained positive for Thy1 while in normal pancreas and chronic pancreatitis cases, values were 11% and 7%, respectively. This has suggested a low background pattern of Thy1 in other tissues compared to PDAC, thus suitable for USMI. Overall, Thy1 neovascular immunostaining could distinguish PDAC from normal and chronic pancreatitis tissues with 90% specificity and 81% sensitivity. We have previously engineered an anti-Thy1-scFv (or simply called “Thy1-scFv”) through directed evolution of scFv protein scaffold using a yeast surface display library^24^. Here, we further engineer Thy1-scFv as a fusion protein with a N-terminal sequence including hexa-histidine tandems (1, 3 or 5 hexa-histidine tags) as purification tags together with a Trx-tag and a S-tag for improving the solubility, and one enterokinase (EK) cleavage site at the junction of Thy1-scFv and the upstream tags. We choose the extensively used Δgor, ΔtrxB, DsbC^+^ SHuffle T7 *E. coli* system to express the engineered protein with disulfide bonds in the cytoplasm. The method we describe here stands out for its relative simplicity to produce Thy1-scFv using complementary tag sequences, which, when cleaved by EK, resulted in generating the native Thy1-scFv without any tag (Fig. 2). Evaluation of native Thy1-scFv showed successful receptor binding capacity in vitro. In vivo results illustrated the conserved functionality of Thy1-scFv enabling PDAC molecular imaging by US. The presented method could be beneficial at the outset of many projects implying eukaryotic protein production and could be optimized toward translational applications.

## Results

### Engineering, expression and purification of Thy1-scFv

#### Construction of vectors expressing Thy1-scFv gene for efficient soluble protein production

**Figure 3:**
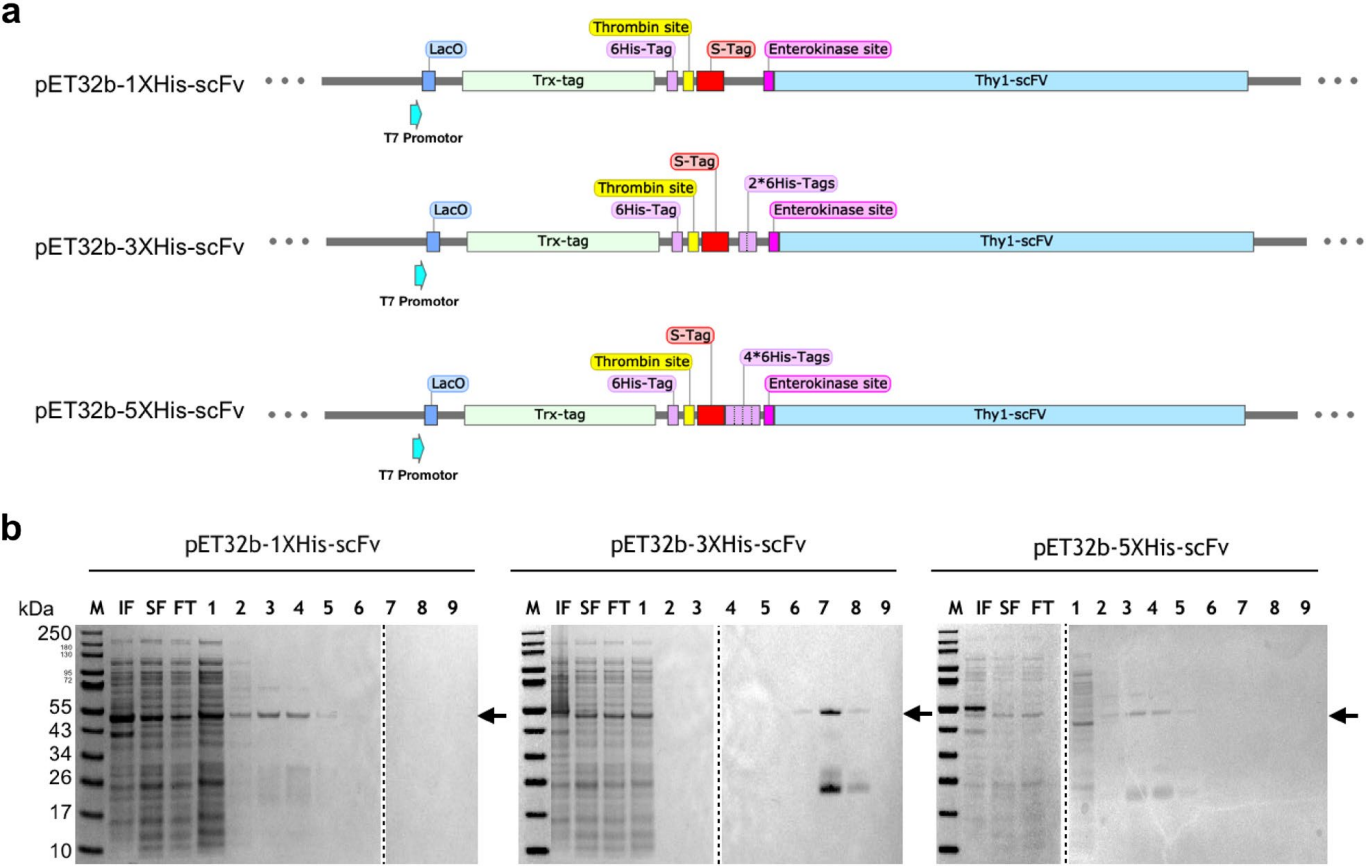
Construction and expression of recombinant Thy1-scFv proteins. (**a**) Schematic map showing the cloning pattern of pET-32b vector constructed to express recombinant Thy1-scFv in various formats (different number of hexa-histidine tags): pET32b-1XHis-scFv, pET32b-3XHis-scFv, and pET32b-5XHis-scFv. (**b**) Purity of protein based on the elution fractions after purification by affinity chromatography: Thy1-scFv formats resolved in 4–12% gradient SDS-PAGE. *M* protein molecular weight marker, *IF* insoluble fraction from cell lysate, *SF* soluble fraction from cell lysate, *FT* flow through, lanes 1–9: eluted fractions; arrows indicate the position of the tagged-Thy1-scFvs. The same methodology was applied for all Thy1-scFv formats and gels were processed in parallel. Dotted lines have been used to delineate different gels. Full-length gels are presented in Supplementary Figure S1.

We constructed three different expression vectors in the popular pET32b vector backbone with Trx- and S-tags and additional tandem His-Tags, giving: pET32b-1XHis-scFv, pET32b-3XHis-scFv, and pET32b-5XHis-scFv (Fig. 3a). Sequence confirmed, clones were transformed into T7 SHuffle *E. coli* cells to investigate the effect of each variant on the expression and purification of Thy1-scFv. In all constructs, the tagged-Thy1-scFv has a theoretical molecular weight around 50 kDa.

#### Optimization of conditions for Thy1-scFv expression and purification by affinity chromatography

**Figure 4:**
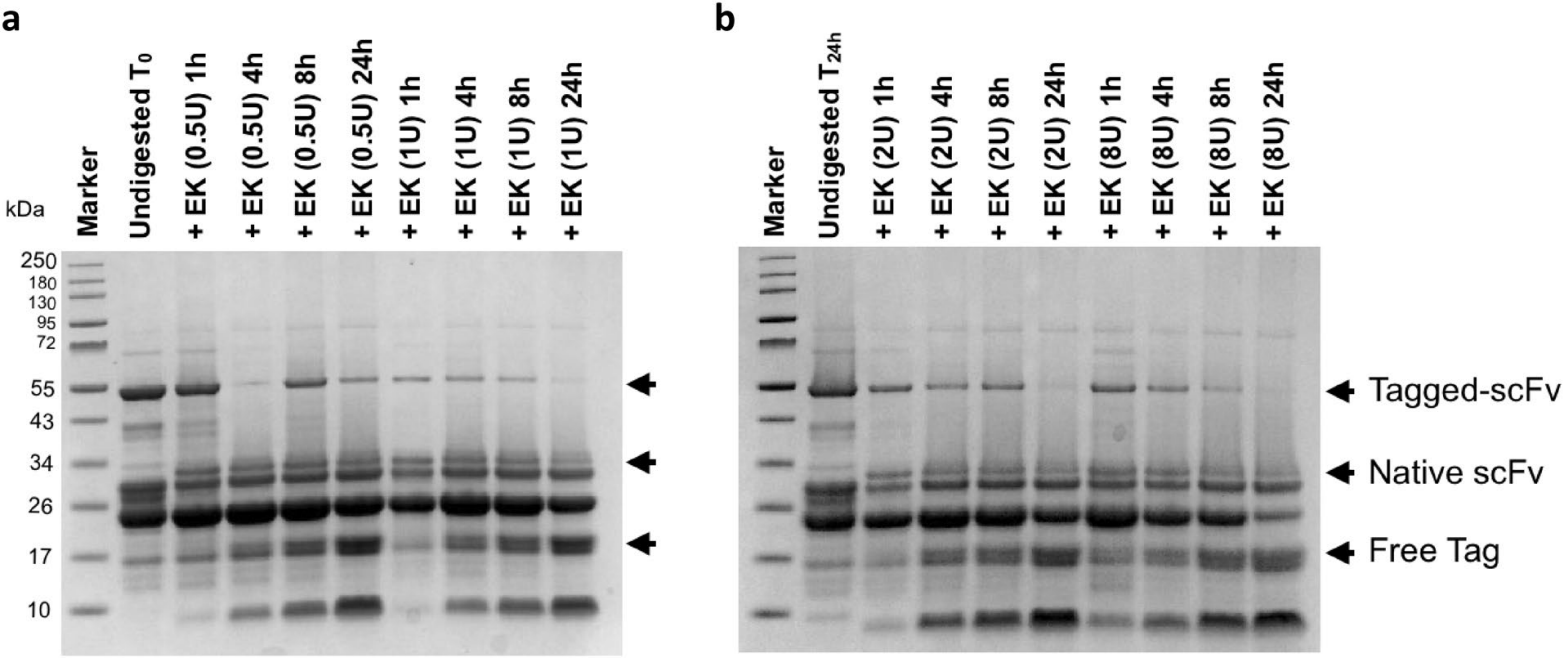
Optimization of EK incubation time and concentrations from (**a**) 0.5U and 1U to (**b**) 2U and 8U, for tag removal of the recombinant Thy1-scFv protein. 4–12% gradient SDS-PAGE were processed in parallel.

Protein expression in *E. coli* was induced at 30 °C (Fig. 3b; Supplementary Figure S1) since higher temperatures usually result in a rapid decrease in protein yield due to degradation and misfolding^25^. As expected, at 37 °C, the expression rate of Thy1-scFv was very low (Supplementary Figure S2), in contrast, we observed an increase in our target protein yield when cultured at 30 °C probably because such lower temperature reduces protein degradation, improves folding efficiency, and thus reduces IB formation. Moreover, endogenous proteases have a higher turnover rate when *E. coli* is grown at 37 °C, thus leading to an enhanced-proteolysis of Thy1-scFv into a Thy1-scFv fragment (around 25 kDa). After purification by affinity chromatography, we detected Thy1-scFv in the elution fractions at an apparent molecular weight consistent with its theoretical mass in all three constructs. Importantly, culture expressing pET32b-3XHis-scFv with an induction temperature of 30 °C showed higher protein level compared to the other constructs (Fig. 3b, pET32b-3XHis-scFv, lanes 6 to 8), and Thy1-scFv was isolated with 47.7 ± 11.5% purity. This is in part due to higher binding of Ni-agarose with the 3X-His-tag used for purification. Apart from the full-length Thy1-scFv, a fragment of around 25 kDa was the major co-purified protein. We suspected it to come from residual endogenous proteolytic activity on Thy1-scFv on a locally weaker structure probably in the multi-histidine tag region. After transfer onto nitrocellulose membranes, an anti-hexa-histidine tag antibody was used to confirm the presence of Thy1-scFv. Analysis of both soluble and insoluble fractions indicates that ~ 35% of the protein can be recovered in the soluble fraction (Supplementary Figure S3). The tagged-scFv from pET32b-1XHis-scFv bound the column with weak affinity and therefore was co-purified with many contaminants. The use of pET32b-5XHis-scFv generated a longer and flexible tag sequence, more prone to influence protein binding sites and improper column binding, as suggested by the affinity chromatography elution profile. Based on these results, the construct pET32b-3XHis-scFv was chosen for further experiments presented in this study. Elution fractions 6–8 from this construct were pooled and used for further enrichment. Elimination of imidazole salts resulted in a more stable protein. The sample was concentrated (Fig. 4, undigested T_0_) yielding to 0.37 ± 0.15 mg of tagged-Thy1-scFv per liter of bacterial culture (Table 1), difficult to reach using other methods of purification.

**Table 1:** Summary of Thy1-scFv purification from 1 L bacterial culture.

Purification step	Volume (mL)	[Proteins]_total_ (mg/mL)	Total proteins (mg)	Tagged-Thy1-scFv purity (%)^a^	Native Thy1-scFv purity (%)^a^	Tagged-Thy1-scFv (mg)	Native Thy1-scFv (mg)
Cell lysate (n = 5)	20	27.2 ± 4.6	543 ± 92	–	–	–	–
IMAC1 (n = 5)	1.1 ± 0.3	0.69 ± 0.1	0.78 ± 0.2	47.7 ± 11	–	0.37 ± 0.1	–
IMAC2 (n = 3)	1.2 ± 1.1	0.15 ± 0.04	0.23 ± 0.1	–	97.7 ± 2.3	–	0.22 ± 0.1

^a^Value based on analysis of Thy1-scFv band intensity using BioRad Gel-Doc system.

#### Optimization of tag removal by EK activity

To optimize the hydrolysis efficacy of EK on the fusion protein, 30 μg of the purified tagged-protein were incubated with 0.5, 1, 2 and 8 Unit(s) of EK at 25 °C for different times (1, 4, 8 and 24 h(s)) (Fig. 4). Incubation of the tagged-Thy1-scFv with EK resulted in the generation of native scFv (~ 30 kDa; expected molecular weight 27,864.93 Da) and free tags (~ 17 kDa). Product bands appeared in all conditions after 1 h incubation along with undigested substrate. After 4 h, the substrate was almost fully digested (approximately 80% of full-length fusion protein was cleaved with 1, 2 and 8 U, Supplementary Figure S4a). However, an incubation time > 4 h or the use of ≥ 8 U of EK during a time > 1 h triggered non-specific cleavage of Thy1-scFv (Supplementary Figure S4b) and was also not cost effective. No spontaneous hydrolysis was detected after 24 h of incubation.

#### Native Thy1-scFv purification

**Figure 5:**
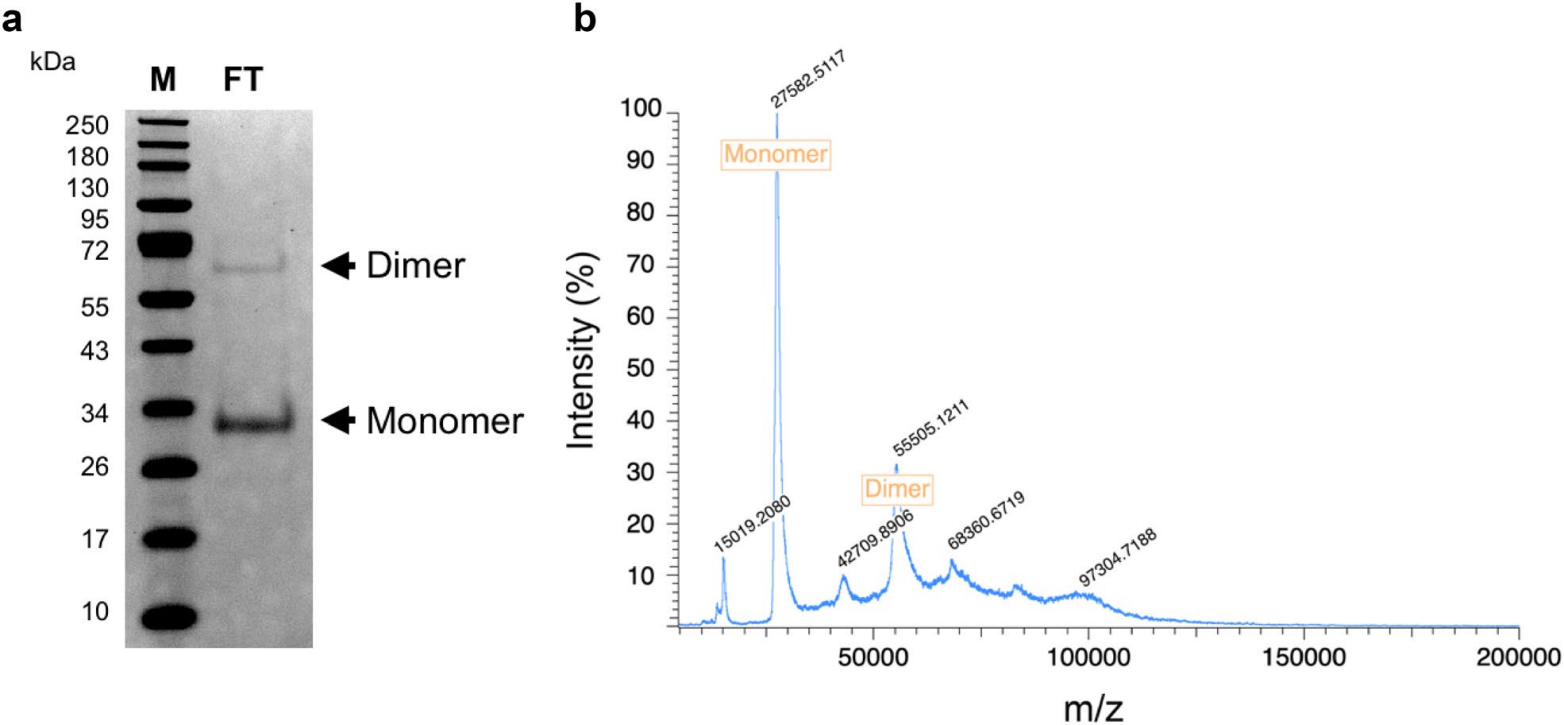
Second step purification of Thy1-scFv after proteolysis. (**a**) SDS-PAGE showing pure Thy1-scFv. *M* protein molecular weight marker, *FT* flow through. (**b**). Mass spectrometry on Thy1-scFv flow through fraction.

**Figure 6:**
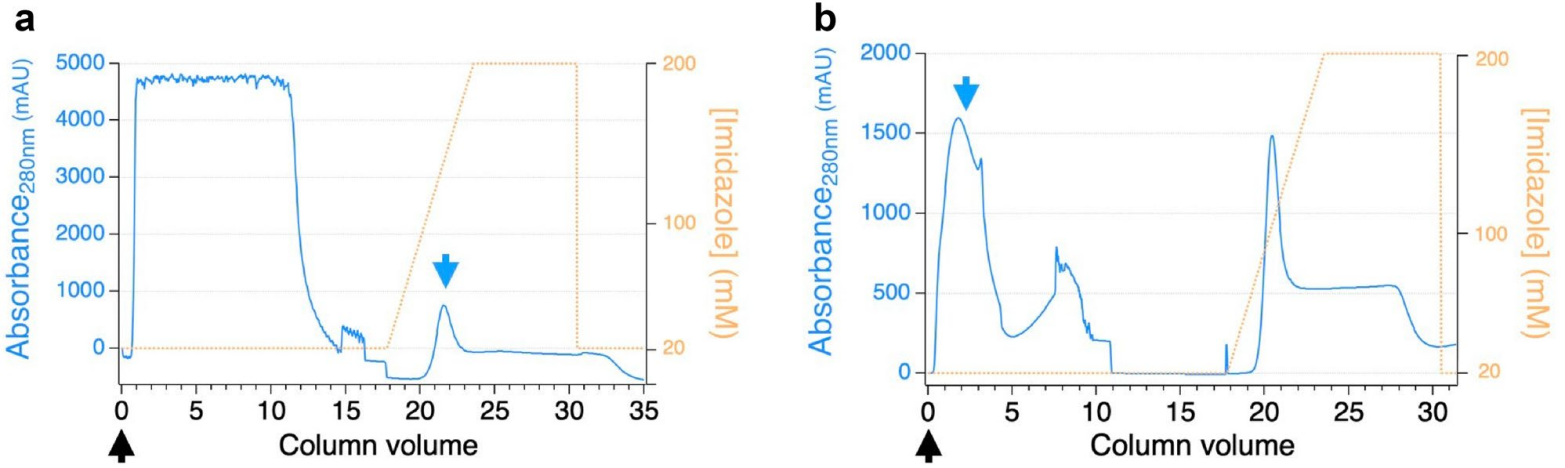
Elution profile of Thy1-scFv on Ni–NTA IMAC. (**a**) IMAC1: tagged-Thy1-scFv is collected during the elution step. (**b**) IMAC2: native Thy1-scFv is collected in the flow through fraction. Black arrows represent the time of injection, and blue arrows indicate the peak and elution time of the protein of interest.

Following a second immobilization by metal affinity chromatography, 0.22 ± 0.11 mg of scFv can be recovered with 97.7% purity as demonstrated by gel electrophoresis (Fig. 5a) and mass spectrometry (Supplementary Figure S5). Further analysis also highlighted a preponderant monomer fraction with a small fraction of dimer (Fig. 5b). The monomer/dimer relative intensity ratio was 10:2 by SDS-PAGE and 10:3 by MALDI analyses. Contrary to the first round of purification (Fig. 6a) where the recombinant protein was recovered in the latest elution fractions, the native protein was isolated in the flow-through fraction (Fig. 6b). Recombinant EK, uncleaved recombinant Thy1-scFv, and fragments containing histidine tags were retained on the column.

### Thy1-scFv binds to Thy1-expressing cells in vitro

**Figure 7:**
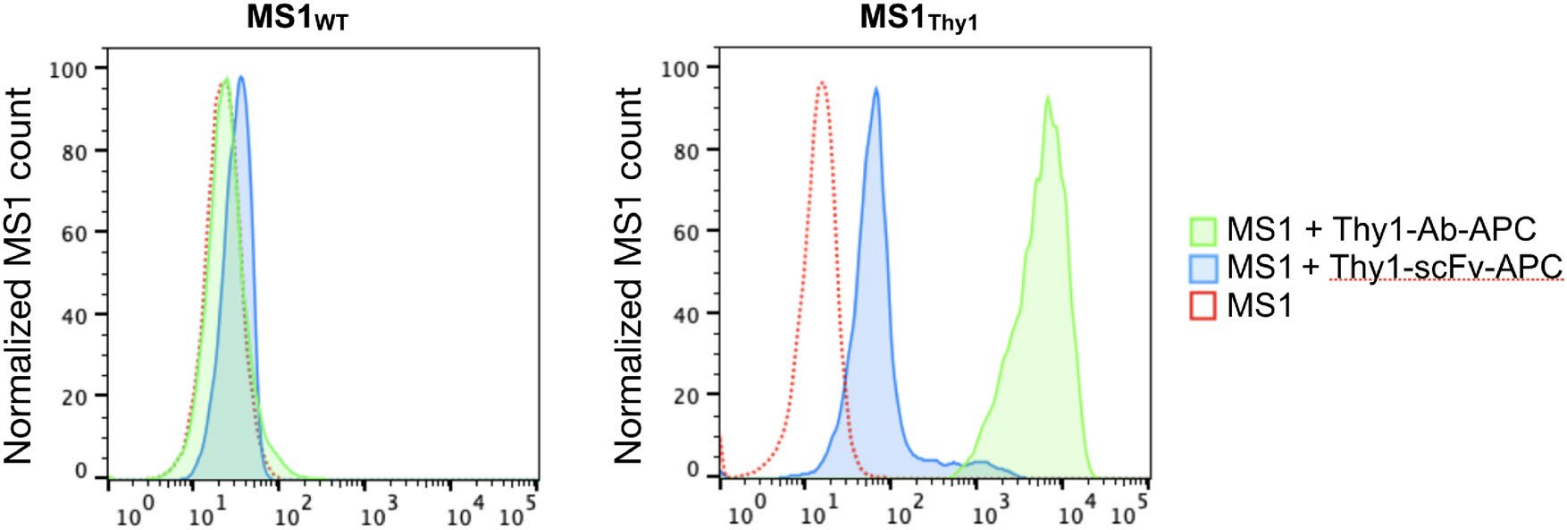
Binding efficiency of Thy1-scFv to MS1 cells.

APC conjugated commercial antibody (Thy1-Ab-APC) was used as positive control to confirm cell-surface expression of Thy1 (Fig. 7). Binding capacity of Thy1-scFv-APC was performed against both Thy1 expressing cells (MS1_Thy1_) and control cells (MS1_WT_) by flow cytometry. Incubation of cells with Thy1-scFv-APC showed higher binding to MS1_Thy1_ compared to the controls as detected by a shift toward increased fluorescent signal intensity. Thus, Thy1-scFv retained its ability to bind to its target cells in vitro*.*

### In vivo US contrast agents functionalized with Thy1-scFv enhance PDAC molecular imaging in transgenic animal model

To test the functionality of Thy1-scFv in vivo*,* we used US contrast agents (MBs) covalently bound with Thy1-scFv on their surface using NHS-chemistry, thus giving MB_Thy1-scFv_. Control MBs, without ligand, were reported as MB_non-targeted_. Prior to imaging, both MB types were tested for size and concentration changes that may occur due to Thy1-scFv’s influence on steric changes or octafluoropropane (C_3_F_8_) gas dissipation.

**Figure 8:**
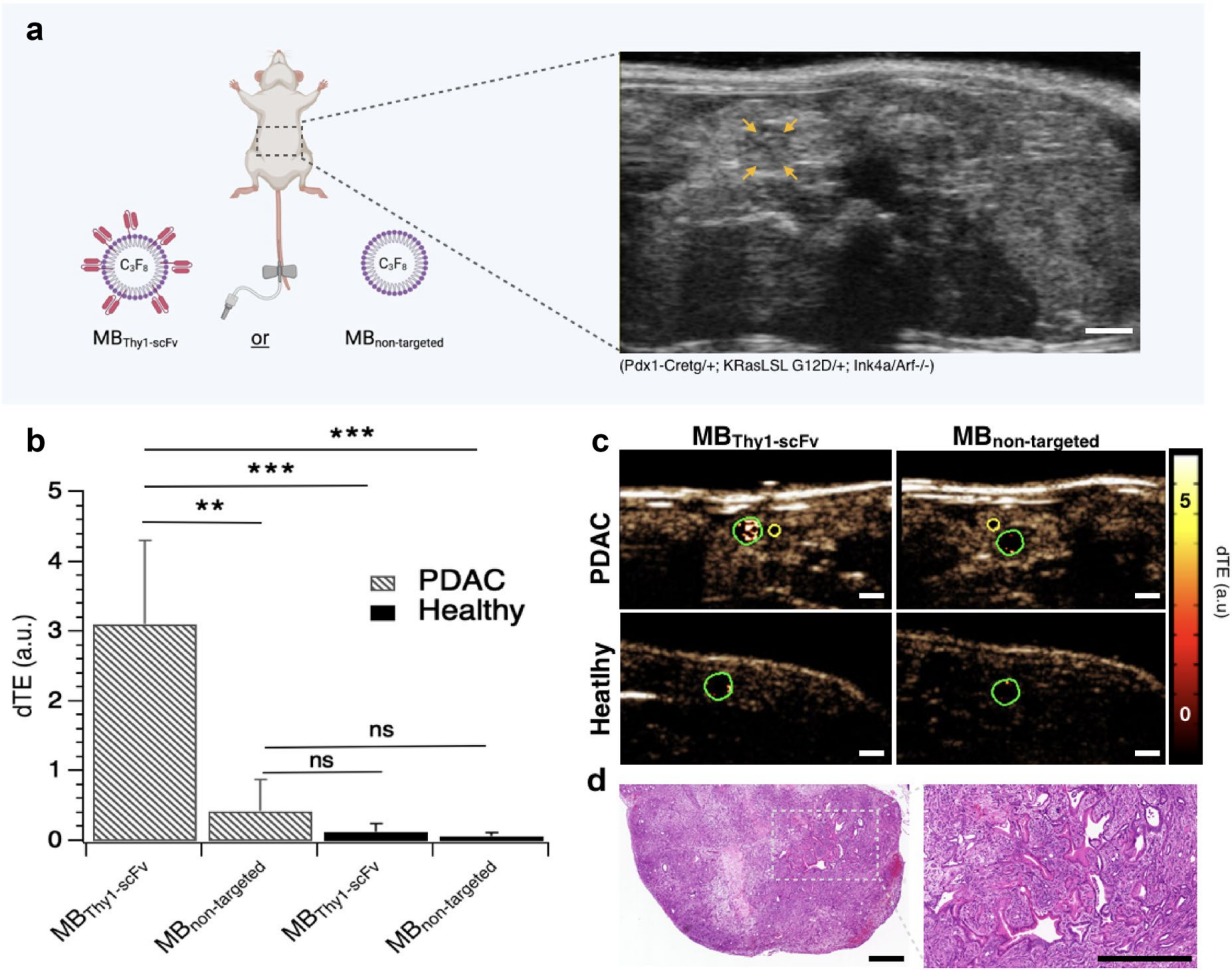
In vivo USMI of Thy1 expression in transgenic mouse model with spontaneous PDAC. (**a**) Schematic illustration of the overall experiment. After localizing the tumor by US B-mode abdominal imaging, MB_non-targeted_ and MB_Thy1-scFv xf_were successively tested for Thy1 binding in PDAC transgenic mice. Orange arrows delineate a pancreatic tumor. B-mode images were used as references to draw region of interest (ROIs) in differential targeted enhancement (dTE) images presented in (**c**). Scale bar 1 mm; (**b**) Quantitative bar graphs of in vivo dTE using targeted and non-targeted contrast agents in PDAC and healthy mice. **p < 0.03; ***p < 0.02; (**c**) Representative transverse dTE images showing stronger signal enhancement in PDAC (green ROI) after injection of MB_Thy1-scFv_, and only low signal following injection of MB_non-targeted_. Background signal was noted in adjacent normal pancreas (a yellow ROI was drawn to quantify imaging signal in adjacent non-PDAC tissue). Scale bar 1 mm; color coded scale is shown for USMI in arbitrary units (a.u.). (**d**) Corresponding hematoxylin–eosin-stained sample confirmed presence of PDAC in transgenic animals. Scale bar 500 μm.

Incorporation of Thy1-scFv as part of the MB composition, *i.e.,* MB_Thy1-scFv_, did not significantly affect the MB mean diameter or size distribution compared to MB_non-targeted_ (mean diameter = 1.2 ± 1.4 μm and 1.1 ± 0.8 μm, respectively) nor the concentration (1.10^9^ particles/mL and 1.2.10^9^ particles/mL, respectively) in agreement with MBs used in the clinic (e.g., Definity (Lantheus Medical Imaging), Sonovue (Bracco Diagnostics)) (Supplementary Figure S6). Tumors were located on B-mode imaging and MBs were injected intravenously starting with MB_non-targeted_ and then MB_Thy1-scFv_ in this specific order (Fig. 8a). On dTE images, mice injected with MB_Thy1-scFv_ showed tumors with significantly increased Thy1 molecular imaging signal (3.1 ± 1.2 a.u.) compared to MB_non-targeted_ (0.4 ± 0.4 a.u.), with a quantitative outcome of ~ 7.8-fold (p < 0.03) (Fig. 8b,c) compared to control. Conversely, imaging of healthy pancreas did not produce any significant differences in imaging signal between the two MB constructs (MB_Thy1-scFv_ = 0.1 ± 0.1 a.u. and MB_non-targeted_ = 0.07 ± 0.06 a.u) and were significantly lower than with MB_Thy1-scFv_ in PDAC model (39-fold, p < 0.02). Histological analysis of H&E-stained tissues confirmed presence of PDAC (Fig. 8d). Overall, these results demonstrate conserved Thy1-targeting property of Thy1-scFv in vivo and sketches the applicability for PDAC USMI.

## Discussion

Molecular imaging has made a considerable contribution to oncology throughout the course of early detection and prognosis, and is an integral part of clinical trials. Biomarkers can be detected using various targeted-probes based on antibodies, peptides or proteins, oligonucleotides, or small molecules conjugated to imaging agents for suitable imaging modalities. Specifically, recombinant protein expression has become an established technique for production of cancer specific antigen-binding ligands in bacterial systems. However, conventional methods can be cumbersome toward meeting criteria for clinical applications. The purpose of this study was to engineer a production model for recombinant protein in their native form through the example of Thy1-scFv, while introducing its potential for early diagnosis of pancreatic cancer.

In vitro refolding technology of IBs has become prevalent to recover insoluble eukaryotic proteins expressed in *E. coli*^10,26^. IBs are usually denatured with high concentration of chaotropes such as urea or guanidine hydrochloride^27^. The major drawbacks of this method are its complex and expensive operational process, which further needs optimization at multiple steps. In addition, use of high concentrations of denaturing agents results in complete denaturation of the secondary structure favoring re-aggregation during successive process^26^. Thus, recovery of soluble and active protein can be greatly reduced. In this study, we proposed an alternative to such extreme procedures and presented a multi-step process for enhanced-soluble protein production in SHuffle T7 *E. coli* cells cytoplasm using gene fusion technology. We used the well-known Trx-tag, improving disulfide bond formation, combined with S-tag. S-tag, commonly used as affinity tag, was here employed to enhance Thy1-scFv solubility thanks to its abundance in charged and polar residues^28^. Both Trx-tag and S-tag are small and do not interfere with the proper folding or function of a fused target protein. Although essential for the expression and purification of recombinant proteins, tag sequences have the potential to interfere with the structure and the function of their fusion partner. In addition, many tags have interacting partners in mammalian systems which can interfere with the biological applicability of recombinant proteins while generating a strong immune reaction. Therefore, tag removal should be considered, especially if the target protein is intended for pharmaceutical or therapeutic clinical applications, for crystallization, and for structural determination studies^15^. Chemical cleavage methods are usually inexpensive^29^ but most systems rely on endopeptidases to separate the fusion partner from the protein of interest^30^. Serine proteases such as the activated blood coagulation factor X (Factor Xa), EK, and thrombin, have been used, as well as viral proteases such as tobacco etch virus (TEV) protease and rhinovirus 3C protease. Viral proteases have a more stringent sequence specificity due to their much slower turnover rates (catalytic rate constant (kcat))^31–35^, however EK has no amino acid specificity requirement on the P’ part of the scissile bond (DDDDK↓) (only proline and tryptophan should be avoided on P1’ which corresponds to an alanine residue in our study). Consequently, when an affinity tag is joined to the N terminus of the protein of interest, EK is able to regenerate a native N terminus. Moreover, we used a recombinant EK presenting the same affinity tag attached to the protein of interest, i.e., hexa-histidine tag. This allowed to apply the digestion products on the same affinity chromatography for separation. Undigested fusion protein substrate, tagged-protease, cleaved tag, and any endogenous proteins that bound to the affinity resin will be separate from the untagged protein of interest in the unbound effluent. After removal of the fusion partner tag, native Thy1-scFv was recovered (0.22 mg ± 0.11) and used for further in vitro and in vivo experiments. We proved its binding functionality in vitro using Thy1-expressing cells. It can be noted that our cell binding assay demonstrated a high shift in fluorescence between our scFv and the corresponding commercial full-length antibody. Knowing that Thy1-Ab-APC (Thermo Fisher, CA) was purchased as such, while Thy1-scFv was conjugated to APC following our own protocol where we used biotin-streptavidin chemistry, the number of dyes per molecules could be different depending on the conjugation chemistry used by the manufacturer. Moreover, full-length antibodies (MW ~ 150 kDa) will likely have more reactive amino acids available than smaller scFvs. For in vivo imaging, we used Thy1-scFv after conjugating to microbubbles by NHS-chemistry. We successfully imaged PDAC neovasculature in transgenic animals. It can be mentioned that a flexible cysteine-tag introduced on the C-terminal end of the scFv could give the possibility for multiple conjugation chemistry while providing site-specific labeling of molecules bearing maleimide functional groups for various applications.

Based on our results, we anticipate that the vector design and basic strategy presented in this study should be applicable to many proteins of biological interest which are currently difficult to purify. Overall, it is generally assumed that for *E. coli*, a 1-L fermentation will generate ~ 150 mg of total cellular proteins. Assuming an average yield between 0.5 and 5% of total proteins, 0.75–7.5 mg of recombinant protein is available in the cells. Based on our results, the protein of interests can be recovered between 30 and 50% in the soluble fraction, hence, our strategy could allow convenient small-scale production of biologically important recombinant proteins to initiate most studies (0.1–3 mg). Scale of production could be expanded by establishing a large-scale fermentation system to produce higher amount of proteins.

Given the low median survival rate (5-year survival rate < 9%) and the low percentage of PDAC patients qualifying for tumor resection (10–20%), the need for early screening methods is globally recognized^36^. Efforts have been made to develop molecular imaging probes capable of detecting early stage PDAC^37^. Here, we produced Thy1-scFv conjugated-US contrast agent, MB_Thy1-scFv_, and illustrated its potential for non-invasively enhancing USMI contrast between PDAC and normal pancreatic tissues in mice consistent with related findings^24,38^. Our probe, able to detect small foci in the pancreas (> 2 mm), constitutes a promising translatable USMI agent. Naturally, the reproducibility and the sensitivity for PDAC molecular imaging will have to be further analyzed*.* With the success of the first and, to date, only targeted-MB in clinical trials for various cancers, BR55 (kinase insert domain receptor-targeted peptide), a rapid expansion of targeted US contrast agents is expected. Our promising pre-clinical results with MB_Thy1-scFv_ could provide opportunities for improved PDAC prognosis, and the presented targeted US contrast agent strategy, a variable format for other biomarker targeting.

## Materials and methods

### Ethical approval

The Administrative Panel on Laboratory Animal Care of Stanford University approved all procedures using laboratory animals used in this study, and all experiments were conducted in accordance with the Guidelines for the Care and Use of Laboratory Animals (APLAC-33828). This study was carried out following the ARRIVE guidelines.

### Expression vector design

The expression vectors pET32b-1XHis-scFv, pET32b-3XHis-scFv, and pET32b-5XHis-scFv were constructed for Thy1-scFv expression. A ligation substrate featuring Thy1-scFv protein was amplified by PCR using a forward primer with NcoI restriction enzyme site and a reverse primer with XhoI restriction enzyme site. The amplified fragment was digested with NcoI and XhoI and ligated into pET-32b(+) prokaryotic expression vector digested with respective restriction enzymes to construct pET32b-1XThy1-scFv with a single inherent hexa-histidine-tag located between the Trx- and S-tags. To introduce more hexa-histidine-tags to construct pET32b-3XHis-scFv and pET32b-5XHis-scFv vectors, we inserted annealed forward and reverse primers coding for 2 and 4 additional hexa-histidine-tags (i.e., 3XHis and 5XHis total) with BglII restriction enzyme site on both the sides as overhangs with 5′-phosphate group. After ligation into pET32b-1XThy1-scFv vector previously digested with BglII restriction enzyme and dephosphorylated using Calf intestine alkaline phosphatase, we generated two additional vectors with 3X and 5X hexa-histidine-tags. All three plasmids contain: a T7 promotor; two fusion partners Trx- and S-tags for enhancing protein folding and solubility; 1, 3 or 5 hexa-histidine tag(s) for purification by immobilized metal affinity chromatography (IMAC); a DDDDK sequence on the N terminus of Thy1-scFv for tag removal using EK cleavage; and the Thy1-scFv gene. Each histidine tag was separated from each other by a few amino acid residues to increase flexible folding. The sequence confirmed, vectors were transformed into SHuffle T7 *E. coli* cells (New England Biolabs, Ipswich, MA) for recombinant protein expression. Oligonucleotides and recombinant protein sequences used in this study for constructing the vectors are listed in Supporting Information (Supplementary Table S1).

### Thy1-scFv expression

Bacterial transformation was performed for each expression vector as follows: 50 μL of SHuffle T7 *E. coli* competent cells were transformed with 1 μg of expression plasmid using standard heat-shock procedure; 300 μL SOC growth media was then added in each vial for cell recovery. Cells were grown at 30 °C for 1 h in a shaking incubator (100 r.p.m.), and plated on Lysogeny Broth (LB)-agar medium containing ampicillin (50 μg/mL). After overnight growth, one fresh-picked colony was inoculated in 2 mL LB-ampicillin medium (50 μg/mL) and grown overnight at 30 °C (250 r.p.m.). Bacteria were transferred into 1 L of LB-ampicillin medium and further cultured until the OD_600nm_ reaches 0.4. The culture was induced for protein expression by the addition of isopropyl-β-d-thiogalactoside (IPTG, 1 mM) after diluting with the addition of one-fourth volume of pre-warmed LB-ampicillin medium. Induction was allowed for 4 h at 30 °C (250 r.p.m.). The pellet was then harvested via centrifugation (5000*g*, 10 min, 4 °C) and stored at − 80 °C.

### Fusion protein purification (IMAC1)

Cell pellets were resuspended in 20 mL of ice-cold lysis buffer (3 mM monosodium phosphate, 50 mM disodium phosphate, 500 mM NaCl, 5% glycerol (v/v), 5 mM CHAPS, and 20 mM imidazole containing protease inhibitors (Thermo Scientific, Rockford, IL)) and lysed by sonication (60% amplitude, 5 s on/off, 10 cycles, Branson SLPe). The soluble and insoluble fractions were then separated by centrifugation (12,000g, 10 min, 4 °C). Insoluble fractions containing cell debris and possible IBs were washed with 8 mL of the same lysis buffer. The soluble fractions were applied to a 1 mL FF His-trap column (GE Healthcare Biosciences, PA) in an AKTA FPLC system (GE Healthcare Biosciences) equilibrated with PBS buffer containing 20 mM imidazole to reduce non-specific binding. The tagged-Thy1-scFv was purified using a linear gradient of imidazole (from 20 to 200 mM) in PBS buffer at a flow rate of 1 mL/min. Concentration of proteins were measured by UV spectrometry in each fraction. Purity of the fractions was analyzed on 4–12% gradient SDS-PAGE followed by staining with Coomassie Blue (SimplyBlue SafeStain, Carlsbad, CA) for visualization using a BioRad Gel-Doc system. Fractions containing the protein of interest were pooled and the best expression vector was utilized for further experiments. Western blot analysis was performed using anti-His tag antibody. A 4–12% gradient SDS-PAGE of the insoluble and soluble fractions was electroblotted onto a 0.2 µm pore size nitrocellulose membrane (Bio Rad, Hercules, CA), blocked in PBS-T (PBS with 0.05% Tween 20) with 5% milk powder for overnight at 4 °C and then treated with anti-His tag antibody (BioLegend, San Diego, CA). After washing, the membrane was incubated with HRP-conjugated anti-mouse IgG antibody. Signals were visualized by the addition of enhanced-chemiluminescence (ECL) substrate and imaging using IVIS in vivo imaging system (Perkin Elmer, Santa Clara, CA).

### Fusion protein cleavage by enterokinase

The purified protein was further treated with EK to remove the overhang tags (trx, S-tag and His-tags). Before EK treatment, the protein solution containing PBS-imidazole buffer was exchanged with 10 mM Tris HCL, 300 mM NaCl, pH7.4, using a 30-kDa molecular weight cutoff Vivaspin Protein Concentrator Spin Column (GE Healthcare Lifesciences, Pittsburgh, PA). In addition, the sample was concentrated during the same step. Tag removal was performed using EK enzyme tagged with hexa-histidine (Genscript, Piscataway NJ; MW = 22.7 KDa) ranging from 10 to 160 IU per milligram of protein during 1 h to 24 h incubation period at 25 °C. Optimal EK concentration and incubation time were used for further experiments.

### Purification of native protein (IMAC2) and characterization for purity

Following EK mediated cleavage, the sample was loaded on the equilibrated 1 mL FF His-trap column. Thereafter, the flow through was collected, followed by the application of a linear gradient of PBS buffer containing imidazole ranging from 20 to 200 mM. The fractions were collected and examined for Thy1-scFv purity and size by Matrix-Assisted Laser Desorption/Ionization time-of-flight (MALDI-TOF) mass spectrometry and SDS-PAGE. Fractions containing the native Thy1-scFv were pooled and further desalted/concentrated using a 10-kDa molecular weight cutoff Vivaspin Protein Concentrator Spin Column (GE Healthcare Lifesciences, Pittsburgh, PA) with PBS buffer, pH 7.4.

### Cell culture

Wildtype MILE SVEN 1 (MS1_WT_) mouse vascular endothelial cells (CRL2279; American Type Culture Collection (ATCC)) and MS1 cells engineered to stably express human Thy1 protein (MS1_Thy1_) (selected using puromycin antibiotic marker) were cultured under sterile conditions in Dulbecco’s Modified Eagle Medium (DMEM) supplemented with 10% FBS and 100 U/mL penicillin and 0.1% streptomycin and maintained in 5% CO_2_ at 37 °C and used for Thy1-scFv binding assays as stated below.

### Binding of Thy1-scFv to Thy1-expressing MS1 cells

To evaluate the biological functionality of Thy1-scFv to specifically bind Thy1-expressing cells, MS1_WT_ and MS1_Thy1_ cells were incubated with Thy1-scFv-APC conjugate. Dye conjugation was performed by biotinylation of Thy1-scFv followed by streptavidin-APC incubation following the manufacturer's recommendation. Briefly, Thy1-scFv was incubated with 20-fold molar excess of NHS-PEG4-Biotin (Pierce Biotechnology, Rockford, IL) at room temperature for 30 min in PBS. Excess of biotin was removed using a Zeba spin column (Pierce Biotechnology, Rockford, IL) before the addition of streptavidin-APC (Tonbo Biosciences, San Diego, CA) to form Thy1-scFv-APC. Then, MS1_WT_ and MS1_Thy1_ cells were incubated with Thy1-scFv-APC (500 nM) or with a commercial Thy1-Ab-APC (eBioscience Inc, San Diego, CA) for 1 h at 4 °C, washed 3 times with PBS. Fluorescence intensity from the dye-labeled ligands were visualized by flow cytometry (Guava easyCyte; Luminex Corp., Austin, TX) and analyzed using FlowJo software (Becton Dickinson & company).

### Synthesis and preparation of MBThy1-scFv as targeted-US contrast agent

Preparation of the US contrast agents, i.e., MBs, is detailed in the Supplementary Materials. Briefly, Thy1-scFv was conjugated to commercial phospholipids by NHS chemistry giving MB_Thy1-scFv_. A non-targeted control, MB_non-targeted_, was made with non-functionalized phospholipids.

### Transgenic mouse model of PDAC

The transgenic pancreatic cancer mouse model (Pdx1-Cre^tg/+^; KRas^LSL G12D/+^; Ink4a/Arf^−/−^) (n = 3), which spontaneously developed foci within 4–7 weeks after birth, was used^39^. Correct genotype was validated for all mice. Tumor diameter ranging between 1.3 and 2.2 mm (mean 1.7 ± 0.4 mm) based on US images were used for the study. Healthy age-matched littermates were used as normal control group (C57BL/6, 6 weeks old, Charles River, Wilmington, MA, n = 3).

### In vivo US molecular imaging of pancreas

In vivo US imaging of vascular Thy1 expression in transgenic PDAC mice and C57BL/6 mice with normal pancreas was performed using two MB constructs (MB_Thy1-scFv_ and MB_non-targeted_) by following the protocol reported previously^24^. In brief, a total of 10^8^ MB_Thy1-scFv_ or MB_non-targeted_ (100 µL) was utilized for intravenous bolus injection via tail vein. All in vivo imaging studies were performed in contrast mode using a dedicated small animal high resolution US imaging system (Vevo 2100, FUJIFILM VisualSonics, Inc., Toronto, ON, Canada) with a linear transducer (MS250, VisualSonics) placed over the abdomen of mice, guided by B-mode imaging to detect the target tissue of interest. Contrast mode images were acquired at 18 MHz, and all imaging parameters (focal length, 10 mm; transmit power, 4%; mechanical index, 0.2; dynamic range, 40 dB) were kept constant during all imaging sessions. A total time of 5 min was allowed for MBs to attach their target before binding quantification. To differentiate the acoustic signal owing to MBs attachment to Thy1 and the signal from freely circulating MBs, the previously described destruction-replenishment technique was employed^22^. The protocol consisted of 3 steps: (1) 200 frames of images capturing blood-vessel bound and unbound MBs within the ROI, (2) a high pressure destructive pulse (1-s continuous high-power destructive pulse of 3.7 MPa, transmit power, 100%; mechanical index, 0.63) to destroy all bound and unbound MBs, and (3) an additional set of 200 frames to measure the signal magnitude from the unbound MBs flowing into the ROI immediately after the destructive pulse. The difference in US imaging signal pre- and post-destruction corresponds to the Thy1 attached contrast agents, MB_Thy1-scFv_ or MB_non-targeted_. A waiting interval of 20 min was maintained between each MB injection to allow for complete clearance before subsequent imaging. Any remaining attached MBs were destroyed by applying a high-power destruction pulse (see above for acoustic parameters).

### Ultrasound molecular imaging data analysis

The molecular imaging signals were quantified post image acquisition with correction for breathing motion artifacts using Vevo 2100 integrated analysis software (VevoCQ; VisualSonics). Data analysis was accomplished by manually drawing ROIs around PDAC tissues, adjacent non-PDAC tissues, as well as in the normal pancreas of control littermates. The magnitude of imaging signal from attached MBs was assessed by subtracting the average imaging signals pre- and post-destruction and expressed as the differential targeted enhancement (dTE) in arbitrary units (a.u.).

### Ex vivo analysis of pancreas tissues

PDAC mice were euthanized in accordance with animal care guidelines. The pancreas was excised and fixed in 4% paraformaldehyde (Santa Cruz Biotechnology Inc., CA) at 4 °C for 24 h. Tissues were cryosectioned and stained with hematoxylin eosin before analysis using a Nanozoomer (Hamamatsu, Japan).

### Statistical analysis

Student-t test was applied to determine statistical significance (**p < 0.03; ***p < 0.02) between groups and data expressed as mean ± standard deviation (S.D.).

## Supplementary Information

Supplementary Information 1.

Supplementary Information 2.

Supplementary Information 3.
