# Supplementary material for: Expression and purification of a native Thy1-single-chain variable fragment for use in molecular imaging
Source: Sci Rep. 2021 Nov 29;11:23026. doi: 10.1038/s41598-021-02445-2 (PMC8630227; doi:10.1038/s41598-021-02445-2)
Supplement: Supplementary file 1 — Supplementary Information 1. [file 41598_2021_2445_MOESM1_ESM.docx]

**S****u****pplementary Materials**

**Expression and Purification of a Native Thy1-Single-Chain Variable Fragment for Use in Molecular Imaging**

Natacha Jugniot, Ph.D.^1^, Rakesh Bam, Ph.D^1^, and Ramasamy Paulmurugan, Ph.D.^1*^

^1^Department of Radiology, Molecular Imaging Program at Stanford, Stanford University, Palo Alto, CA, USA.

**Supplementary Methods**

***Recombinant Thy1-scFv sequences***

- **Trx-1XHistag-Thrombin-Stag-EK- Thy1-scFv**

MSDKIIHLTDDSFDTDVLKADGAILVDFWAEWCGPCKMIAPILDEIADEYQGKLTVAKLNIDQNPGTAPKYGIRGIPTLLLFKNGEVAATKVGALSKGQLKEFLDANLAGSGSGHMHHHHHHSSGLVPRGSGMKETAAAKFERQHMDSPDLGTDDDDKAMASASQVQLVQSGAEVKKPGASVKVSCKASGYTFTGYYVHWVRQAPGQGLEWMGWVNPNSGDTNYAQKFQGRVTMTRDTSISTAYMELSGLRSDDTAVYYCARDGDEDWYFDLWGRGTPVTVSSGILGSGGGGSGGGGSGGGGSDIRLTQSPSSLSASIGDRVTITCRASQGISRSLVWYQQKPGKAPRLLIYAASTLQSGVPSRFSGSGSGTDFTLTISSLQPEDFATYYCLQHNTYPFTFGPGTKVDIKSGIPEQKLGGGGGC

- **Trx-3XHistag-Thrombin-Stag-EK- Thy1-scFv**

MSDKIIHLTDDSFDTDVLKADGAILVDFWAEWCGPCKMIAPILDEIADEYQGKLTVAKLNIDQNPGTAPKYGIRGIPTLLLFKNGEVAATKVGALSKGQLKEFLDANLAGSGSGHMHHHHHHSSGLVPRGSGMKETAAAKFERQHMDSPDL**HHHHHHGDLHHHHHH**GDLGTDDDDKAMASASQVQLVQSGAEVKKPGASVKVSCKASGYTFTGYYVHWVRQAPGQGLEWMGWVNPNSGDTNYAQKFQGRVTMTRDTSISTAYMELSGLRSDDTAVYYCARDGDEDWYFDLWGRGTPVTVSSGILGSGGGGSGGGGSGGGGSDIRLTQSPSSLSASIGDRVTITCRASQGISRSLVWYQQKPGKAPRLLIYAASTLQSGVPSRFSGSGSGTDFTLTISSLQPEDFATYYCLQHNTYPFTFGPGTKVDIKSGIPEQKLGGGGGC

- **Trx-5XHistag-Thrombin-Stag-EK- Thy1-scFv**

MSDKIIHLTDDSFDTDVLKADGAILVDFWAEWCGPCKMIAPILDEIADEYQGKLTVAKLNIDQNPGTAPKYGIRGIPTLLLFKNGEVAATKVGALSKGQLKEFLDANLAGSGSGHMHHHHHHSSGLVPRGSGMKETAAAKFERQHMDSPDLHHHHHHGDLHHHHHHGDLHHHHHHGDLHHHHHHGDLGTDDDDKAMASASQVQLVQSGAEVKKPGASVKVSCKASGYTFTGYYVHWVRQAPGQGLEWMGWVNPNSGDTNYAQKFQGRVTMTRDTSISTAYMELSGLRSDDTAVYYCARDGDEDWYFDLWGRGTPVTVSSGILGSGGGGSGGGGSGGGGSDIRLTQSPSSLSASIGDRVTITCRASQGISRSLVWYQQKPGKAPRLLIYAASTLQSGVPSRFSGSGSGTDFTLTISSLQPEDFATYYCLQHNTYPFTFGPGTKVDIKSGIPEQKLGGGGGC

Trx Sequence

HHHHHH: 1XHis-Tag

LVPRGS: Thrombin Sequence

KETAAAKFERQHMDS: S-Tag

(**HHHHHHGDLHHHHHH: 2XHistag**)

(HHHHHHGDLHHHHHHGDLHHHHHHGDLHHHHHH: 4XHistag)

DDDDK : EK Sequence

ScFv Sequence

GGGGGC : linker for site-specific labelling

| Primer Sequence |
| --- |
| **F-Histag:** PGATCTCCACCATCATCATCATCATGGA |
| **R-Histag:** PGATCTCCATGATGATGATGATGGTGGA  **F-scFv:** CCATGGCGAGCGCGAGCCAGGTGCAAC  **R-scFv:** CTCGAGTTAGCAACCGCCACCGCCACCCAGTTTTTGTTCCGGAAT |

**Table S1.** PCR primers for cloning pET32b-1XHis-scFv, pET32b-3XHis-scFv, and pET32b-5XHis-scFv.

***Synthesis and preparation of MB_Thy1-scFv_ as targeted-US contrast agent***

The phospholipids (1,2-dipalmitoyl-sn-glycero-3-phosphate (DPPA); 1,2-dipalmitoyl-sn-glycero-3-phosphocholine (DPPC); 1,2-distearoyl-sn-glycero-3-phosphoethanolamine-N-methoxypolyethylene glycol)-5000 (DSPE-MPEG(5000)] (Avanti Polar Lipids, Inc., Alabaster, AL) were weighted and saline was added. Phospholipids were solubilized and homogenized using our LV1-microfluidic system (Microfluidics, Westwood, MA). We set the microfluidic system at 30,000 psi, washed the system working-track with 75% ethanol solution twice, then re-washed with saline three times. We injected the phospholipid mix into the microfluidic system, and extracted the solubilized solution at the outlet. This process was repeated three times for complete solubilization. The phospholipid (1,2-distearoyl-*sn*-glycero-3-phosphoethanolamine-N-[succinimidyl(polyethylene glycol-5000)] (DSPE-PEG(5000)-NHS) (Nanosoft Polymers, Winston-Salem, NC) was weighted separately, dissolved in chloroform, and vacuumed for 4h. The dried lipid film was then dissolved in 0.1 M sodium bicarbonate buffer, pH 7.4, prior to the addition of Thy1-scFv (10:1 molar ratio) for lipid functionalization at 4°C for 16h. Phospholipids used for MB_Thy1-scFv_ were composed of DPPA, DPPC, DSPE-MPEG(5000), and DSPE-PEG(5000)-NHS at a 7:55:1:4 molar ratio, and phospholipids composing MB_non-targeted_ contained DPPA, DPPC, and DSPE-MPEG(5000) at a 7:55:5 molar ratio. A non-ionic copolymer surfactant, Pluronic F-127 (0.03 mg/mL; Sigma-Aldrich, St. Louis, MO) was dissolved with glycerol (125 mg/mL; Sigma-Aldrich, St. Louis, MO) and propylene glycol (105 mg/mL; BioWorld, Dublin, OH) in saline in a separate container. The emulsifier solution and each phospholipid solution were then mixed and distributed in 3 mL glass vials (Wheaton, Millville, NJ). The vials were sealed before filling with octafluoropropane gas (Fluoromed, L.P., Round Rock, TX). Vials were agitated by a Vialmix (Lantheus Medical Imaging, Inc., North Billerica, MA) for 45 s to form the MBs. Vials were centrifuged (4°C; 300 g, 3 min). MBs were purified and concentrated by replacing the lower aqueous solution with 0.5 mL of sterile physiological saline. The solutions were gently agitated and washed 2 additional times using the same procedure. Concentration and size distribution of MB_Thy1-scFv_ and MB_non-targeted_ were determined using an AccuSizer Model 770A (Particle Sizing Systems, Inc., Santa Barbara, CA, USA).

**Supplementary Results**

**
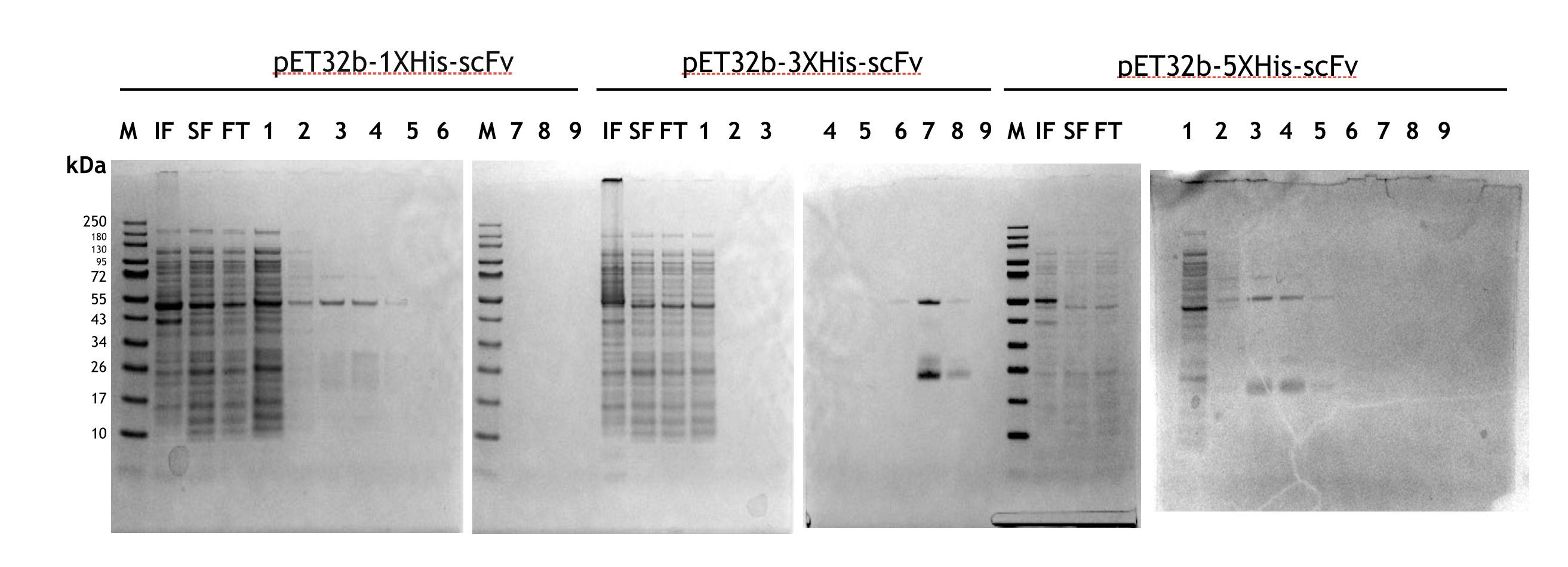
**

**Figure S1.** Full-length protein gels of Thy1-scFv purification. The same methodology was applied for all Thy1-scFv formats and gels were processed in parallel. M: protein molecular weight marker; IF: insoluble fraction from cell lysate; SF: soluble fraction from cell lysate; FT: flow through; lanes 1-9: eluted fractions.


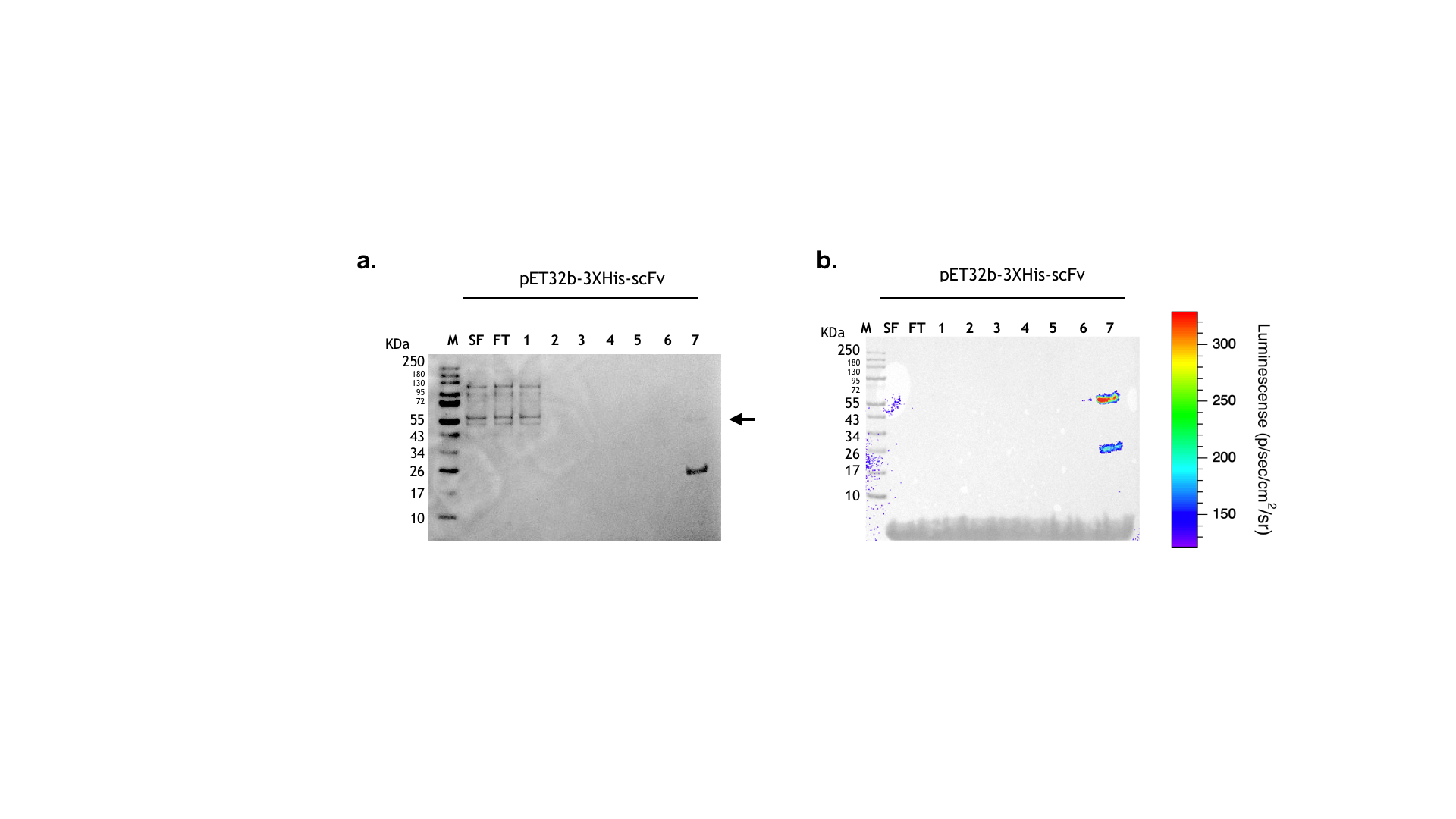


**Figure S2.** Thy1-scFv expression from pET32b-3XHis-scFv expression vector in *E. Coli* induced at 37°C. **(a)** Elution profile of Thy1-scFv. The black arrow indicates the position of the tagged-Thy1-scFv. (**b)** Western blotting showing the presence of Thy1-scFv in elution 7 together with a Thy1-scFv fragment. M: protein molecular weight marker; SF: soluble fraction from cell lysate; FT: flow through; lanes 1-9: eluted fractions.

**Figure S3.** Solubility of Thy1-scFv variants. **(a)** Recovery of Thy1-scFv in the soluble and insoluble cell lysate fractions by western blotting. **(b)** Quantitative analysis of Thy1-scFv. The same methodology was applied for
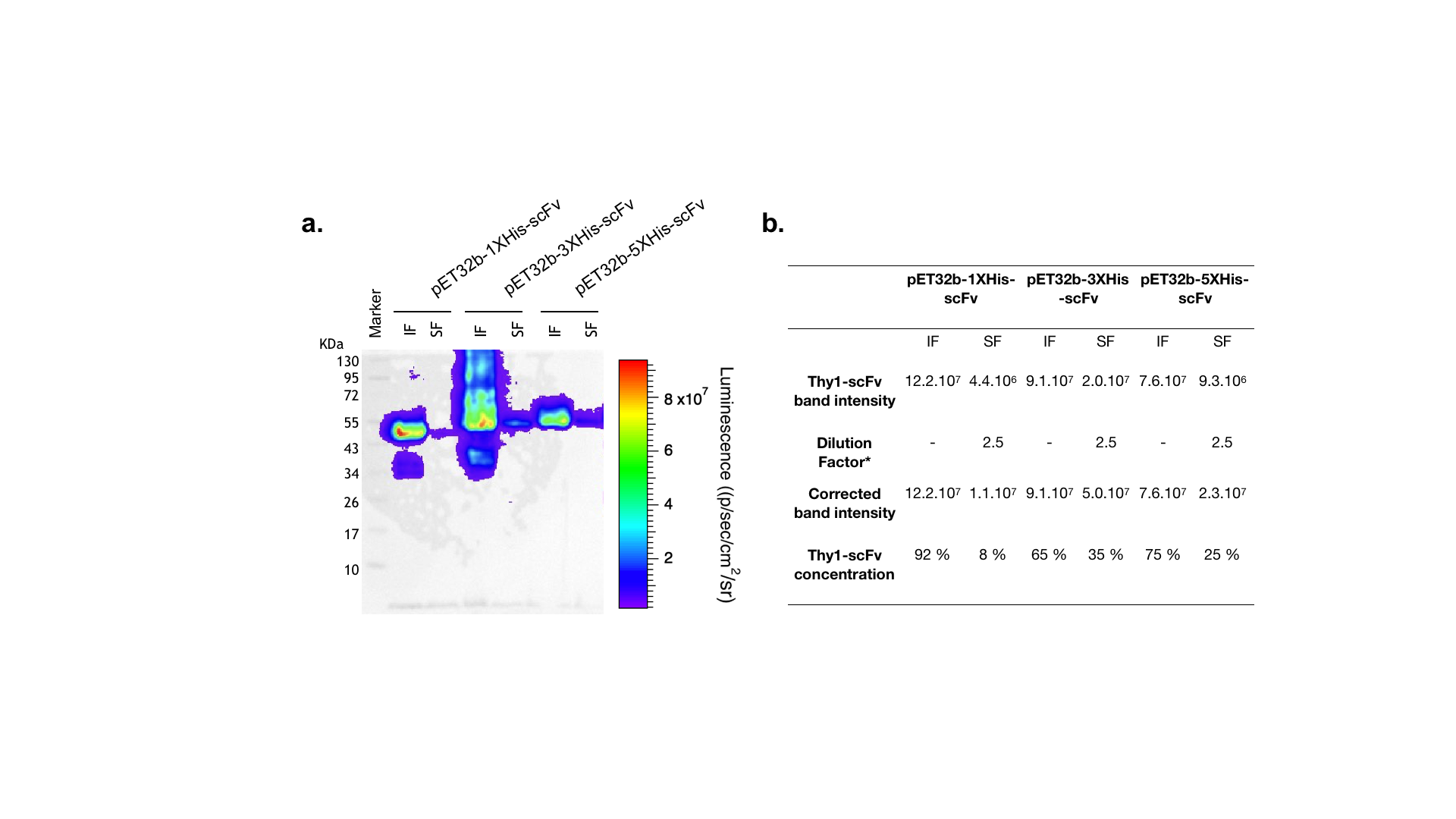
 all Thy1-scFv formats and samples were processed in parallel. *Values of luminescence have been corrected by a dilution factor applied on the soluble fraction and equal to 2.5. Marker: protein molecular weight marker; IF: insoluble fraction from cell lysate; SF: soluble fraction from cell lysate.

**Figure S4.** Proteolysis of tagged-Thy1-scFv by EK. **(a)** Tagged-Thy1-scFv consumption over 24 hours; **(b)** Native Thy1-scFv formation; **(c)** Free tag sequence formation; Intensity values were evaluated based on protein gels using BioRad Gel-Doc system.


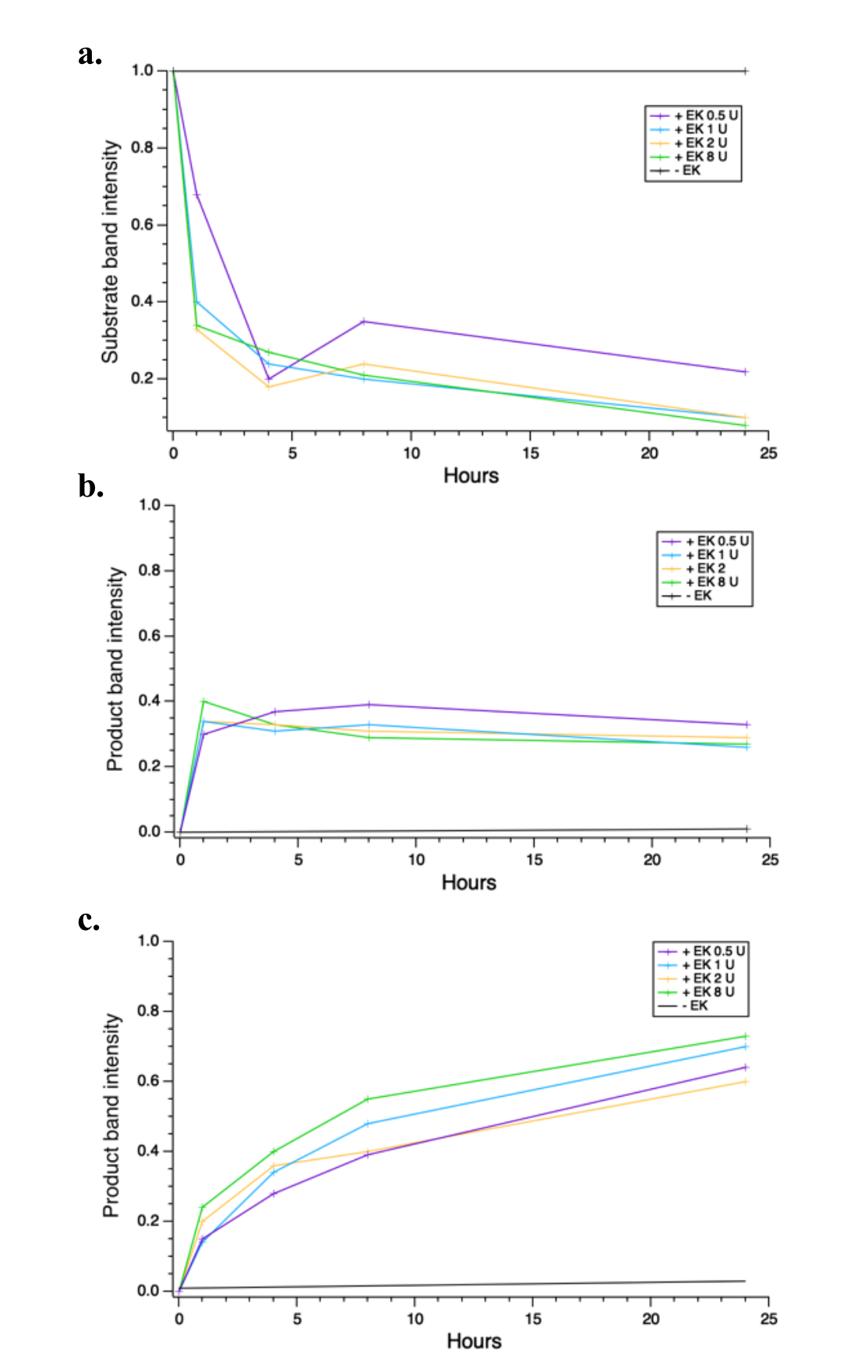


Time (hours)

Time (hours)

Time (hours)

Product2 band intensity (a.u)

Product1 band intensity (a.u)

Substrate band intensity (a.u)

Thy1 (monomer)

Thy1 (dimer)

BSA


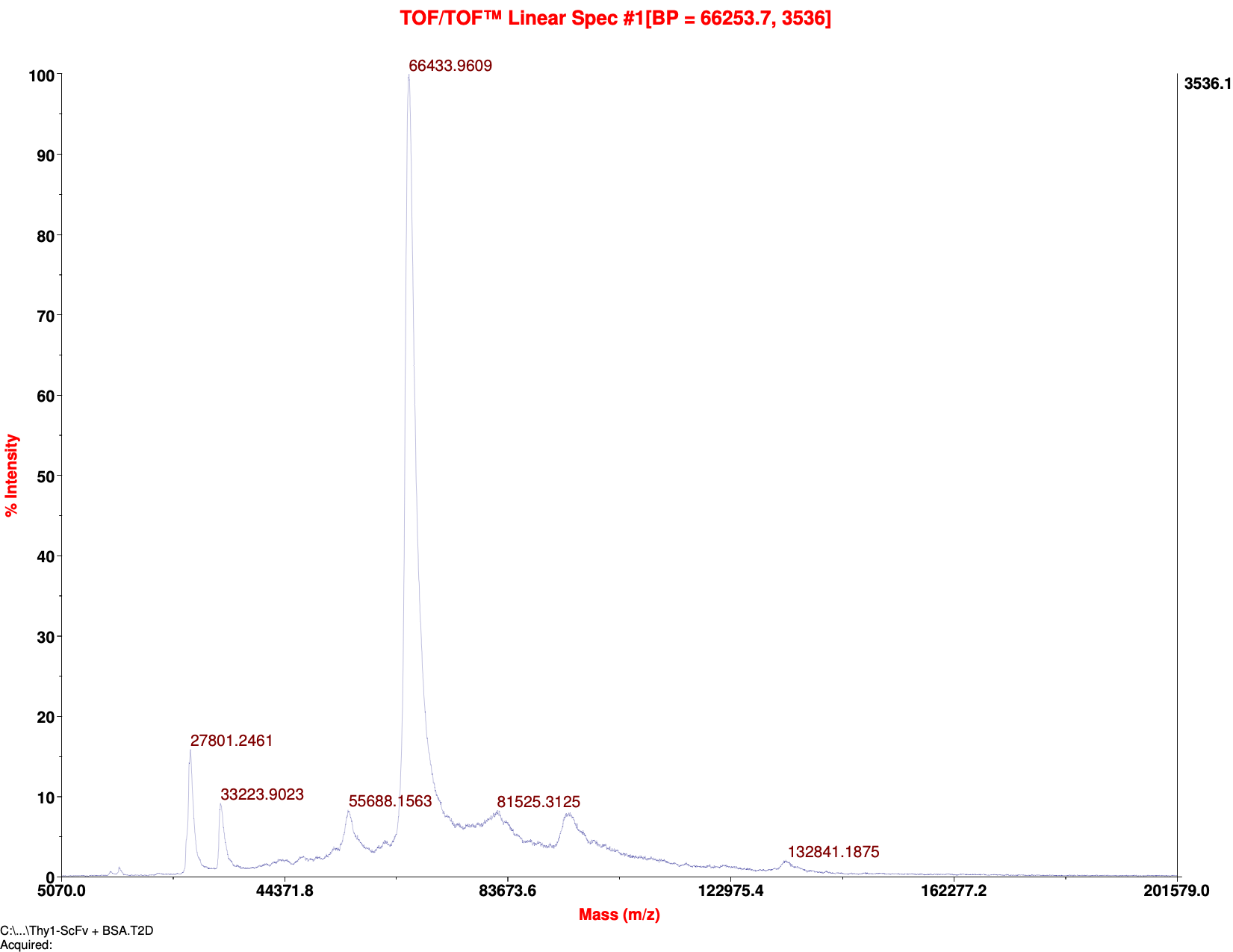


**Figure S5.** Mass spectrum of native Thy1-scFv with bovine serum albumin (BSA) as internal standard (66433.9609 Da). Sample ran with BSA as internal standard gave better accuracy with an observed mass of Thy1-scFv monomer only 63 Da away from its theoretical mass (27801.24 Da *versus* 27864.93 Da, respectively) and a 85 Da difference for the dimer (55688.15 Da *versus* 55602.48 Da, respectively).


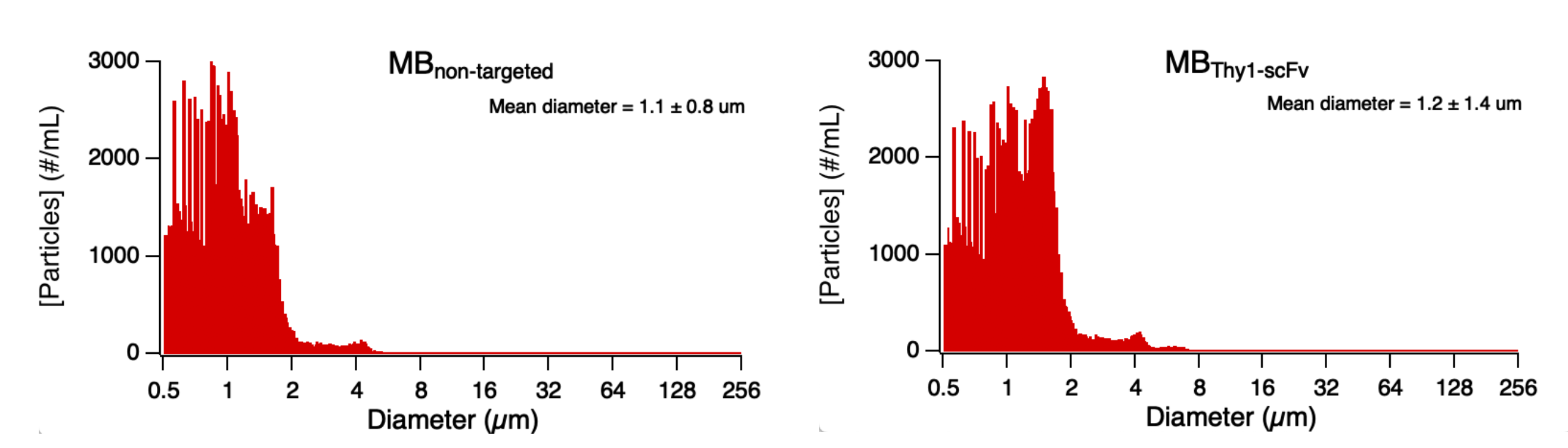


**Figure S6.** Diameter size characterization of MB_non-targeted_ and MB_Thy1-scFv_.
